# Audits of Antimicrobial Usage in a Tertiary Care Center in Hyderabad

**DOI:** 10.7759/cureus.21125

**Published:** 2022-01-11

**Authors:** Lakshmi Jyothi, Ariyanachi K, Saranya M, Chennakesavulu Dara, Varatharajan Sakthivadivel, Triven Sagar Sandepogu, Archana Gaur

**Affiliations:** 1 Microbiology, All India Institute of Medical Sciences, Bibinagar, Bibinagar, IND; 2 Anatomy, All India Institute of Medical Sciences, Bibinagar, Bibinagar, IND; 3 Microbiology, Employees' State Insurance Corporation (ESIC) Medical College and Hospital, Hyderabad, IND; 4 Medicine, Employees' State Insurance Corporation (ESIC) Medical College and Hospital, Hyderabad, IND; 5 Internal Medicine, All India Institute of Medical Sciences, Bibinagar, Bibinagar, IND; 6 Internal Medicine, Employees' State Insurance Corporation (ESIC) Medical College and Hospital, Hyderabad, IND; 7 Physiology, All India Institute of Medical Sciences, Bibinagar, Bibinagar, IND

**Keywords:** surgical prophylaxis, antibiotic misuse, culture reports, antibiotic prescribing, prescription auditing

## Abstract

Background

Irrational prescriptions have an ill effect on health as well as on healthcare expenditure. Prescription auditing is an important tool to improve the quality of prescriptions, which in turn improves the quality of health care provided. Regular and timely audits of antibiotic prescriptions can prevent irrational antibiotic usage.

Introduction

The inappropriate use of drugs is a global health problem, especially in developing countries like India. In 2015, during the 68th World Health Organization (WHO) Regional Committee for Southeast Asia, all Member States of the region, including India, endorsed the “Regional Strategy for Patient Safety in the WHO Southeast Asia Region (2016-2025)” aiming to support the development of national quality of care and patient safety strategies, policies, and plans and commit to translating those objectives of the Regional Strategy into actionable strategies at country level.

Methodology

A retrospective observational study was conducted in a 330-bedded, National Accreditation Board for Hospitals & Healthcare Providers (NABH)-accredited tertiary healthcare center. The study period was six months, from January 2019 to June 2019.

Results

Ninety-five point four-five percent (95.45%) of the doctors attended the sensitization program and all accepted following the standard prescribing protocols. Sixty-nine point seven percent (69.7%) of the doctors were aware of the availability of drugs in the hospital pharmacy stores. Seventy-four point two-four percent (74.24%) of the doctors were aware of the ongoing prescription audits. Seventy-two point two-seven percent (72.27%) of the treating doctors were of the opinion of selecting the appropriate antibiotics based on hospital antibiogram. The importance of antibiograms from cultures and environmental surveillance was followed well only after sensitizing all the treating doctors. Ninety-five point four-five percent (95.45%) of the doctors were of the opinion of taking the permission of a higher authority to start high-end antibiotics. Seventy-seven point one-zero percent (77.10%) doctors recommended sample collection prior to antibiotic administration. Sixty-three percent (63%) of the patient’s clinical condition improved with the antibiotics prescribed prior to the culture report.

Conclusion

By judicious use of antibiotics, we can reduce the evolution of antibiotic resistance in bacteria and extend the useful life of antibiotics that are still effective. Antibiotic use patterns must be studied to address complications resulting from a large number of antibiotics.

## Introduction

Irrational prescriptions have an ill effect on health as well as on healthcare expenditure. Prescription auditing is an important tool to improve the quality of prescriptions, which in turn improves the quality of health care provided. Regular and timely audits of antibiotic prescriptions can prevent irrational antibiotic usage. Microbiologists practicing the reporting of minimum inhibitory concentrations (MICs) and minimum bactericidal concentration (MBC) find that they are excellent predictors of the potency of an antimicrobial against the infecting organism, but they provide essentially no information on the time course of antimicrobial activity. MIC provides no information on growth inhibitory effects that may persist after antimicrobial exposure. Hence, it mandates a follow-up regarding the ongoing treatment and response to the treatment without recurrence of symptoms and with minimal side effects.

The inappropriate use of drugs is a global health problem, especially in developing countries like India. Health care systems in India are divided into public and private sectors; treatment in the private sector is much more expensive than in the public sector [1]. Dispensing medications in India is subject to the Pharmacy Act 1948. Registered pharmacists and physicians can dispense medication to patients in India. Medication safety has been recognized as one of the key important components of quality of care and many initiatives have been taking place at central and state levels to address diverse issues of patient safety; like many developing countries, there was no medication safety system or program [2]. In 2015, during the 68th World Health Organization (WHO) Regional Committee for Southeast Asia, all Member States of the region, including India, endorsed the “Regional Strategy for Patient Safety in the WHO Southeast Asia Region (2016-2025)” aiming to support the development of national quality of care and patient safety strategies, policies, and plans and commit to translating those objectives of the Regional Strategy into actionable strategies at country level. In this context, the Ministry of Health and Family Welfare (MoHFW), Government of India, constituted a multistakeholder Patient Safety Expert Group in August 2016. In 2005 the MoHFW in India initiated the National Pharmacovigilance Program (NPP), coordinated by the Central Drugs Standard Control Organization (CDSCO), based in the national capital, New Delhi. This new program was established because of the failure of earlier attempts at pooling ADR reports on a national scale [3].

In India, health expenditure accounts for around 1.4% of the gross domestic product (GDP). In India, there are public and private healthcare systems; private-sector treatment is significantly more expensive than public-sector treatment. The Pharmacy Act of 1948 governs the dispensing of pharmaceuticals in India, where only licensed pharmacists and physicians are permitted to distribute medicine to patients. In 2017, India released its National Action Plan to Combat AMR (NAPAMR), which was in accordance with the worldwide action plan to combat AMR. Antibiotic policies are frequently implemented as part of an antibiotic stewardship program to reduce needless antibiotic usage and improve management. It is necessary to evaluate data on antibiotic prescription trends in patients in order to establish such a policy for infection management. Self-medication with antibiotics is common in India and the prevalence ranges between 16% and 85% in medical students [4-5]. Antibiotics are very important medicines, and they play an important role in reducing morbidity and mortality. Hence, thorough knowledge of antibiotics' prescription patterns and the judicious use of antibiotics is essential to overcome the antibiotic resistance crisis, therefore, increasing the awareness of physicians about this issue is very important.

## Materials and methods

A retrospective observational study was conducted in a 330-bedded National Accreditation Board for Hospitals & Healthcare Providers (NABH)-accredited tertiary healthcare center. The study period was six months, from January 2019 to June 2019.

The aim of this audit was to determine the effect of the awareness of good prescribing practices; evaluate adherence to antibiotic policy; and promote documentation of adverse events.

Audit criteria

The following checklist was chosen for auditing antibiotic usage (Table 1).

**Table 1:** Checklist chosen for auditing antibiotic usage

1	Doctors’ opinion and practice on the prescription pattern of antibiotics as mandated by NABH
2	Common antibiotics prescribed in the hospital
3	Clinical conditions with antibiotic misuse
4	The most frequent types of infections & appropriateness of therapy
5	Whether patient’s clinical condition improved to antibiotics prior to culture
6	Misused antibiotics in the hospital
7	Antibiotics used for surgical prophylaxis
8	Surgical antibiotics prophylaxis time
9	Number of days of administering antibiotics for inpatients
10	Common presentations where empirical antibiotic therapy was required
11	Prescriptions with the generic name and the brand names

The following steps were implied:

a) Sensitised all the clinical practitioners prescribing antibiotics to patients as mandated by NABH. Sensitization programs on prescription audits using the hospital's WhatsApp group chats, weekly meetings for all doctors and nurses department-wise, and recommendations and amendments done as per the accreditation policy.

b) Feedback forms collected from the treating doctors and the residents. Sixty-six doctors filled the forms as mandated by the Accreditation Committee.

c) Awareness of the availability of generic drugs and Food and Drug Administration (FDA)-approved drugs in the store is emphasized.

d) Hospital infection control bulletin released quarterly was always updated on recent updates on the drugs, banned drugs, and fixed doses released by the FDA and the Central Drugs Standard Control Organization.

e) Collected data on antibiotic prescribing prior to the culture and after culture reports.

An effective awareness program on antibiotics usage was conducted regularly for junior doctors and the nursing staff. Nurses maintained the checklist for medication errors, near misses, transcription errors, preparation or dispensing errors, administration errors (wrong patient, dose, time, medication, route of administration, rate, and extravasations (maybe an adverse drug reaction (ADR)), and unauthorized dose given). Further monitoring and sensitization were done for the availability of medications in the hospital pharmacy. We, from the Hospital Infection Control Committee (HICC), were monitoring the availability of antibiotics, storing the drugs at appropriate temperatures according to manufacturer’s instructions. This was followed as per the hospital committee and pharmacotherapeutic committee recommendations. The checklist was prepared for near-expiry drugs in the next three months that were returned to the pharmacy, drugs at appropriate temperatures according to the manufacturer’s instructions, drugs reconstitution and administering, use of drugs left over in the syringes, and finally discarding the left-over drugs as per the biomedical waste rule.

## Results

**Figure 1:**
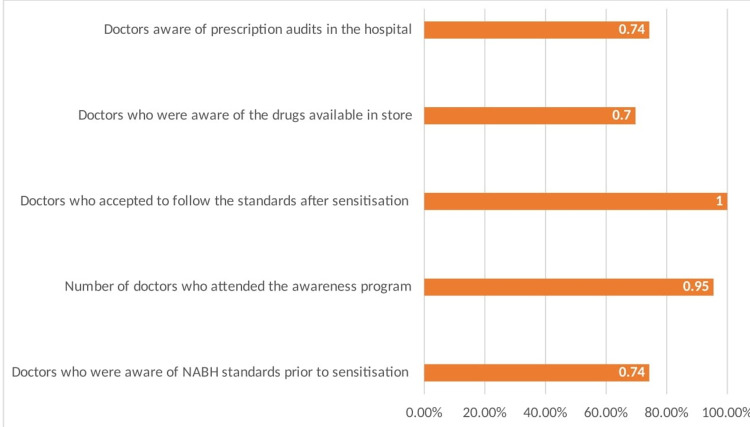
Survey of the awareness of prescribing antibiotics

Clinical practitioners who give antibiotics to patients were asked to fill out a survey on how well they understood the accrediting board's antibiotic prescription audit (Figure 1).

Ninety-five point four-five percent (95.45%) of the doctors attended the sensitization program, and all accepted to follow the standard prescribing protocols. Sixty-nine point seven percent (69.7%) of the doctors were aware of the availability of drugs in the hospital pharmacy stores. Seventy-four point two-four percent (74.24%) of the doctors were aware of the ongoing prescription audits.

**Figure 2:**
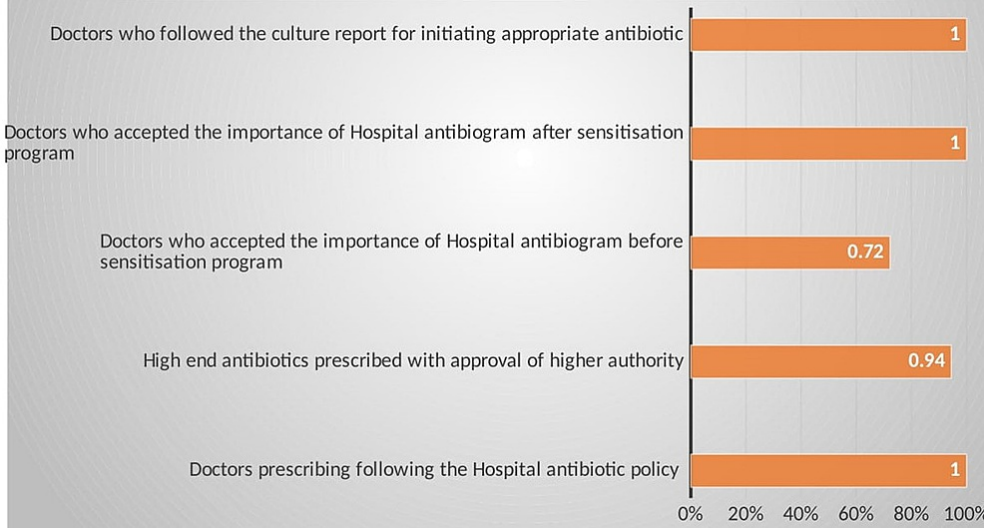
Doctors' antibiotic prescription practices

The treating clinicians were asked to complete a questionnaire on their antibiotic prescribing practices (Figure 2).

One-hundred percent (100%) of the treating doctors have given their recommendations for a proper culture and sensitivity report, and treat accordingly. Seventy-two point two-seven percent (72.27%) of the treating doctors were of the opinion of selecting the appropriate antibiotics based on hospital antibiogram. The importance of antibiograms from cultures and environmental surveillance was followed well only after sensitizing all the treating doctors. Ninety-five point four-five percent (95.45%) of the doctors were of the opinion of taking the permission of a higher authority to start high-end antibiotics.

**Figure 3:**
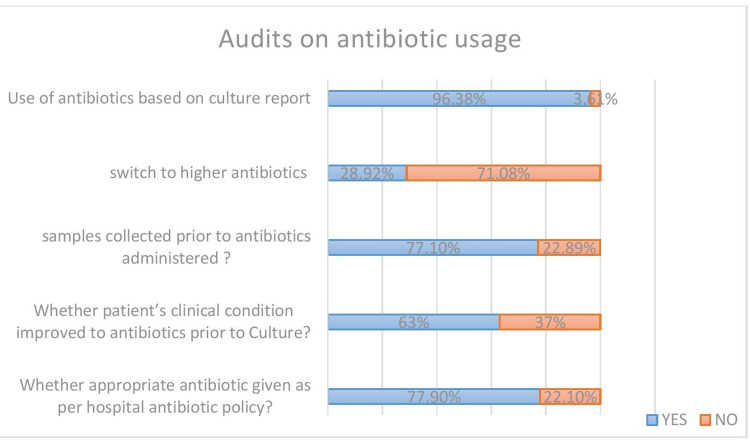
Prescription audits of the importance of culture reports and following the hospital's antibiotic policy

Feedback was received on the significance of culture reports in antibiotic prescribing (Figure 3).

Seventy-seven point nine percent (77.9%) of the prescriptions followed therapy according to the hospital antibiotic policy. Seventy-seven point one-zero percent (77.10%) of doctors recommended sample collection prior to antibiotic administration. Sixty-three percent (63%) of the patient’s clinical conditions improved with the antibiotics prescribed prior to the culture report. Of the 22.1% of the doctors who didn’t follow the hospital policy, 9.2% were more of viral infections, and the rest were high-end antibiotics without the approval of a higher authority.

**Figure 4:**
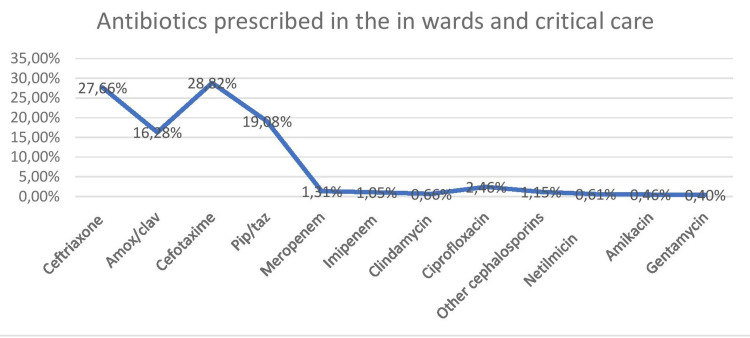
Commonly used antibiotics in the hospital

A survey was done to find out which antibiotics were the most commonly utilized in the hospital (Figure 4).

Antibiotics prescribed in the hospital were analyzed from 3893 prescriptions. Cephalosporins were frequently prescribed, cefotaxime was the commonest followed by ceftriaxone, pip/tazo, and amox/clav. Among the aminoglycosides prescribed, Netilmicin was commonly used followed by amikacin and gentamycin. The commonest injectable used was piperacillin/tazobactam, amox/clav A, imipenem, and amikacin.

**Figure 5:**
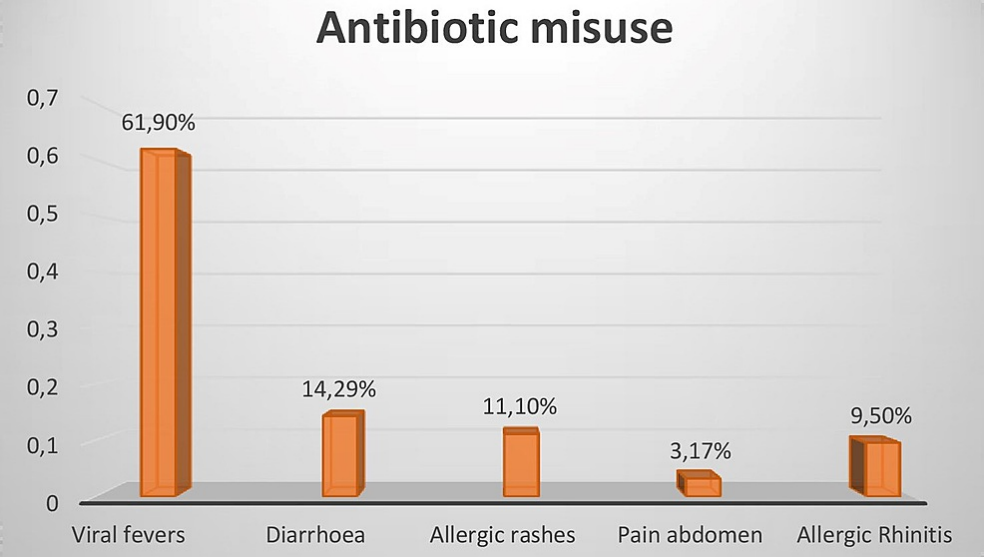
Clinical conditions with antibiotic misuse

A survey was conducted to determine the most prevalent clinical circumstances in which antibiotics were administered inappropriately (Figure 5).

A total of 63 cases were noted; 61.9% of cases presented with viral fevers, and for these cases, further observation was not done, instead, antibiotics were started immediately. Fourteen point two-nine percent (14.29%) of diarrheal cases, 11.1% of allergic rashes, 9.5% of rhinitis cases mostly consisted of the pediatric population and were initiated with first and second-generation cephalosporins; 3.17% of patients presented with pain abdomen. Sensitization on antibiotic usage by the hospital accreditation board mandated observing and starting an antibiotic only if there was no improvement.

**Figure 6:**
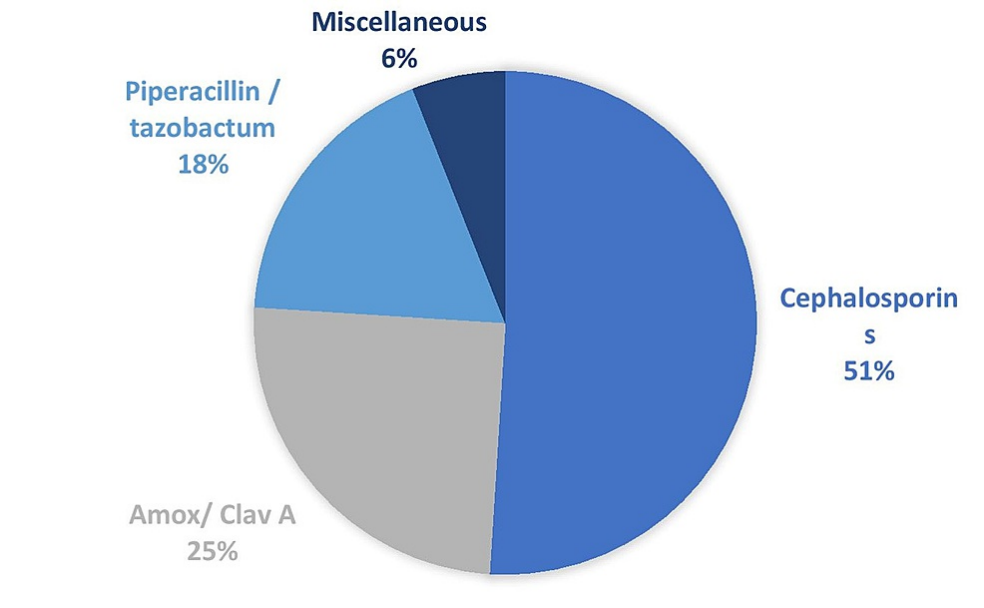
Misused antibiotics in the hospital

The most mismanaged drugs in hospitals were studied (Figure 6). The most misused antibiotics were cephalosporins followed by amox/clav A.

**Figure 7:**
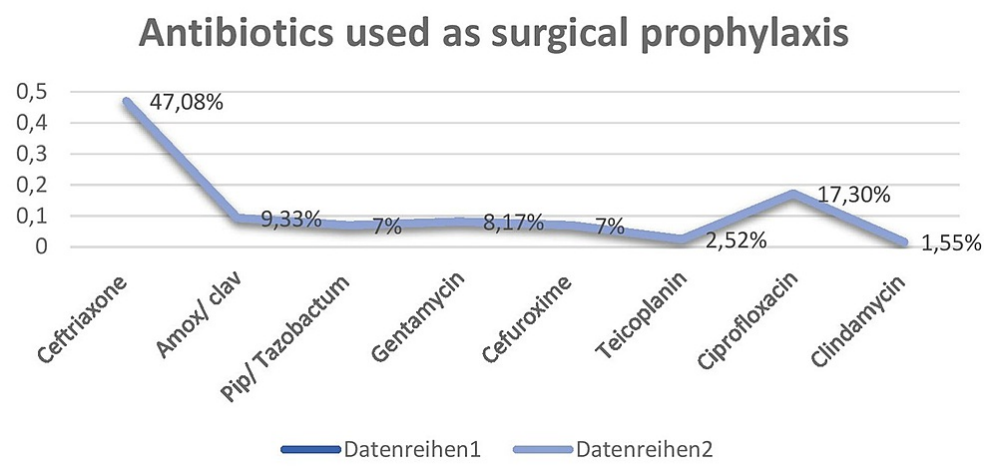
Antibiotics used for surgical prophylaxis

A survey looked at antibiotics often used in hospitals for surgical prophylaxis (Figure 7).

The total number of surgeries in the last six months was 585 (major surgeries) and 379 (minor surgeries). The total number of antibiotics prescribed as antibiotic prophylaxis was 514 cases, which includes 499 major surgeries and 15 minor surgeries. Eighty-six major surgeries were lower segment Cesarian section (LSCS), and prophylaxis was given only after cord clamping. The most common antibiotic used as surgical prophylaxis were cephalosporins, with ceftriaxone - 242 (47.08%), followed by ciprofloxacin - 89 (17.30%), amox/clav- 48 (9.33%), gentamycin - 42 (8.17%), pip/tazobactam - 36 (7%), cefuroxime - 36 (7%), teicoplanin - 13 (2.52%), and clindamycin - 8 (1.55%). Amendments were done after surgical site infections in orthopedic surgeries, where two cases showed growth of methicillin-resistant *Staphylococcus* species, and hence the surgeon preferred to add teicoplanin as a prophylactic drug to prevent surgical site infections in all major orthopedic cases.

**Figure 8:**
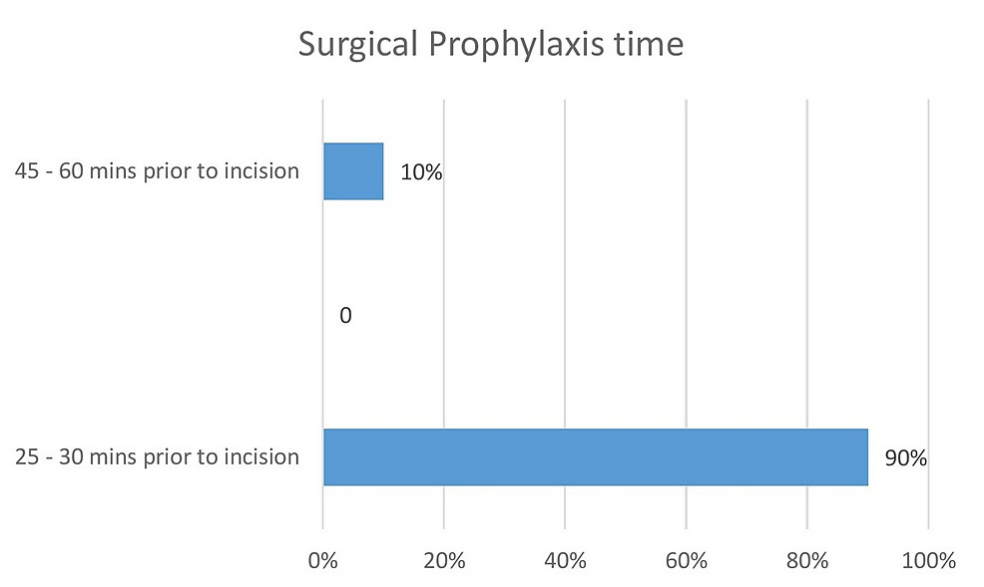
Surgical antibiotics prophylaxis time

A survey of doctors was undertaken to ascertain the amount of time surgical prophylaxis was given before surgery (Figure 8). This was 25 to 30 mins (90%) and 45 mins to one hour (10%).

All the cases were given a test dose 24 hours prior. Most of the surgical cases received prophylaxis within 25 to 30 mins prior to skin incision. The checklist did not include LSCS since the prophylaxis was given after cord clamping.

**Figure 9:**
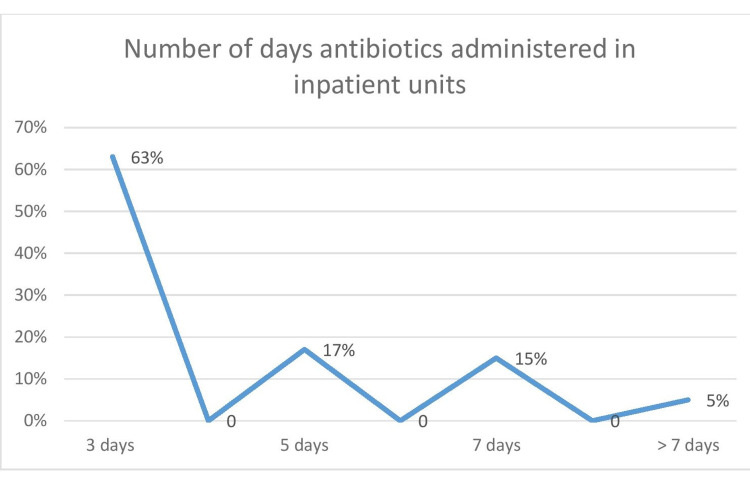
Number of days of administering antibiotics for the inpatients

The number of days an antibiotic is prescribed for a full course of treatment for patients was obtained (Figure 9). Mostly, the antibiotics were given for a period of three days, followed by five days.

**Figure 10:**
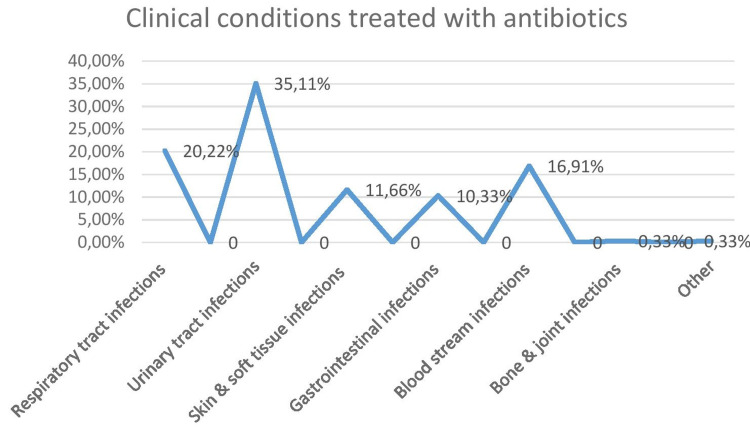
Common presentations where empirical antibiotic therapy was required

The most common clinical conditions seen while initiating empirical antibiotic therapy were evaluated (Figure 10).

The most common presentation was urinary tract infections followed by respiratory tract infections, bloodstream infections, and skin and soft tissue infections where antibiotics were used.

**Figure 11:**
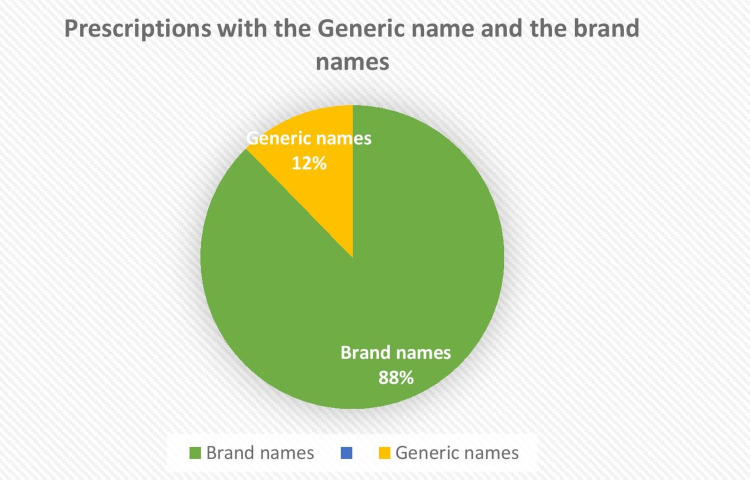
Prescriptions with generic names and brand names

A poll was performed to find out if doctors prefer to prescribe generic or branded antibiotics (Figure 11). Brand names constituted 87.7 % of cases and generic names 12.3%.

Clinicians were mostly prescribing brand names, with very few prescriptions showing the generic name. The brands available in the pharmacy stores were those prescribed by the treating doctors after the approval of the hospital pharmacotherapeutic committee and infection control team (for antibiotics). This policy was also approved by the accreditation committee.

## Discussion

Antibiotics prescribing by physicians has become increasingly important across the world, owing to a rise in antibiotic use, illness prevalence, and drug resistance. Antimicrobials have played a key role in decreasing morbidity associated with the infectious complications of surgical operations, organ transplants, and cancer treatment, in addition to treating isolated episodes of infection. The global growth in bacterial resistance that is threatening the efficacy of antibiotics and antimicrobial resistance (AMR) is increasingly recognized as a serious public health issue [6-7]. Only recently has there been a rise in awareness of AMR in underdeveloped nations.

In India, health expenditure accounts for around 1.4% of GDP. In India, there are public and private healthcare systems; private-sector treatment is significantly more expensive than public-sector treatment. The Pharmacy Act of 1948 governs the dispensing of pharmaceuticals in India. In India, only licensed pharmacists and physicians are permitted to distribute medicine to patients. In 2017, India released its National Action Plan to Combat AMR (NAPAMR), which was in accordance with the worldwide action plan to combat AMR [8]. Antibiotic policies are frequently implemented as part of an antibiotic stewardship program to reduce needless antibiotic usage and improve management. It is necessary to evaluate data on antibiotic prescription trends in patients in order to establish such a policy for infection management [9].

Antibiotic prescription variation within and between nations is a growing topic that needs to be carefully addressed. This variance may suggest poor clinical management, which raises the patient's risk of adverse outcomes, wastes healthcare resources, and contributes to the societal rise of resistant strains [10-11]. According to an estimate, India's crude mortality rate from infectious causes is at 416.75 deaths per 100,000 people [7]. The early detection of illness and initiation of remedial treatment for such infections is critical. Antibiotics are the most often prescribed and abused medications by both patients and doctors. Antimicrobial resistance is on the rise in India, thanks to a high prevalence of infectious diseases, poor living conditions, and cheap access to medications. Antibiotic prescriptions are audited on a regular basis to verify that they are being used appropriately and in accordance with WHO standards [12].

India is the country that consumes the most antimicrobials. Additionally, antibiotic usage is causally connected to the formation of antimicrobial resistance [13]. A proper guideline on the judicial use of antibiotics has to be formulated. Inputs on antibiotic prescription trends among the patients are essential in order to develop an antibiotic strategy, and this study was undertaken for that purpose.

Antibiotic prescription recommendations are provided at the national and state levels as well as in many cases in particular hospital advice papers. Regional and global specialists form groups to develop regional standards. The corporate sector's faulty prescription of antibiotics has been repeatedly documented in the developing world [14]. In our study, 95.45% of the doctors attended the sensitization program and all accepted to follow the standard prescribing protocols. Sixty-nine point seven percent (69.7%) of the doctors were aware of the availability of drugs in the hospital pharmacy stores. Seventy-four point two-four percent (74.24%) of the doctors were aware of the ongoing prescription audits. Only 77.9% in our study were given an appropriate antibiotic as per the hospital antibiotic policy. Antibiotic prescription is influenced by a number of factors such as patient’s socio-economic level, age, associated co-morbidities, and physician characteristics, such as academic qualifications, competence, experience, and source of continuing education; and working environment. To create strategies that will successfully enhance the use of antibiotics, it is vital to study physicians' prescription behavior.

In our study, though all opined that antibiotics should be started after culture sensitivity, only 72.27% of doctors agreed to commence the antibiotic treatment based on the result of the hospital antibiogram. Ninety-five point four-five percent (95.45%) of the doctors were of the opinion of taking the permission of higher authority to start high-end antibiotics. Sixty-one point nine percent (61.9%) of the antibiotics were prescribed only on the basis of clinical diagnosis of viral fever, 14.29% were prescribed on the clinical diagnosis of diarrhea, 11.1% were prescribed on the clinical diagnosis of allergic rashes, 9.5% were prescribed on the clinical diagnosis of rhinitis cases, and only 37% of the patients showed improvement in clinical conditions when antibiotics were prescribed without culture sensitivity. Before administering an antibiotic, the worldwide recommendation is to collect microbiological samples from hospitalized patients with suspected illnesses. In fact, starting antimicrobial therapy too soon can inhibit bacterial growth and prevent the establishment of a microbiological diagnosis, which is necessary for setting a focused antibiotic therapy [15]. In a study done in South India, of 230 physicians surveyed, 51% responded to the use of culture and antimicrobial susceptibility testing before prescribing antibiotics [16]. Culture sensitivity would help prescribe appropriate and rational drugs to the patients rather than prescribing only on the basis of clinical diagnosis. The most successful strategy of regulating antimicrobial usage has been found to be an antimicrobial restriction, either by policy restriction or by requiring preauthorization and rationale [17].

In our study, out of all the patients who were prescribed antibiotics, only 91.8% of them actually needed them. Nine point two percent (9.2%) were given antibiotics, which could have been avoided as these patients were ruled out to have viral fever, streptococcal pharyngitis, or runny nose. If unnecessary antibiotic usage is reduced, resistance is reduced as a result. In a study on macrolide-resistant *Streptococcus pyogenes*, it was demonstrated clearly how reducing macrolide use might lead to a reduction in AMR. Antibiotic resistance has decreased from 9.2% in 1997 to 7.4% in 2000, with a statistically significant link between regional macrolide resistance and consumption rates [18]. Antibiotic prescriptions in URTI patients are reported to be approximately 50% [4,19-20] in studies from all over the world. In a study done by Chua et al., one out of seven participants received inappropriate antibiotics, and that’s just the antibiotics that could be told were inappropriate based on the associated diagnosis. They were satisfied with only 13% of the prescriptions [21]. In another study, acute respiratory conditions per 1000 population led to 221 antibiotic prescriptions (95% CI, 198-245) annually, but only 111 antibiotic prescriptions were estimated to be appropriate for these conditions [22].

Several methods have been recommended to minimize the overuse of antibiotics in upper respiratory tract infections (URTI) patients. Educational materials for physicians, audits and feedback, educational meetings, changes in the financial and healthcare systems, reminders, technological support systems, patient-target interventions, and multidimensional physician-target interventions are the most researched approaches. Studies have shown that interactive educational teachings and physician targeted interventions could reduce inappropriate antibiotic prescription by 20% [23]. In a study done to implement clinical guidelines (CG) to decrease the inappropriate prescription of antibiotics, it was observed that the prescription rate of antibiotics was significantly reduced by 24.5% and the appropriate antibiotic prescription rate was significantly increased by 44.2% in the first post-implementation cohort [24].

In our audit, the most prescribed antibiotics are cephalosporins (56.48%) followed by a beta-lactam /lactamase inhibitor combination (19.08%). Quinolones and carbapenems were less frequently used. In a study done by Remesh et al. [25], beta-lactams were most prescribed (60%). In another study, beta-lactam antibiotics (61.54%), sulphonamides (26.05%), and fluoroquinolones (6.97%) were the preferred drugs [26]. This might be owing to their year-round availability. In a study done by Kaur et al. [9], the commonly prescribed antibiotic was ceftriaxone (69%) For empiric management of infections, it may be necessary to use broad-spectrum antimicrobials initially. Prudence may still be used, such as using amoxicillin rather than co-amoxiclav, limiting the use of third and fourth-generation cephalosporins, and lowering fluoroquinolone usage, all of which have been proven to help reduce antimicrobial resistance.

In this audit, surgical prophylaxis was given for 87.86% of cases (499 major surgeries and 15 minor surgeries) posted for minor and major surgeries in the last six months. The commonly prescribed antibiotics for surgical prophylaxis were cephalosporins, with Cceftriaxone - 242 (47.08%), followed by ciprofloxacin - 89 (17.30%), followed by amox/clav - 48 (9.33%), gentamycin - 42 (8.17%), pip/tazobactam - 36 (7%), cefuroxime - 36 (7%), teicoplanin - 13 (2.52%), clindamycin - eight (1.55%). And the surgical prophylaxis of antibiotics was given to 90% of the patients 25-30 minutes prior to the procedure, and 10% received them 45 minutes to one hour prior to the procedure. Antibiotics used before surgery have been found to reduce the risk of surgical site infections (SSIs). Antibiotic prophylaxis is used to guarantee that the medication is present in effective serum and tissue levels throughout the length of the procedure. A study of 2,847 patients having clean or clean-contaminated surgical procedures found that those who received antibiotic prophylaxis within two hours of incision had a 0.6% surgical site infection incidence. Patients who received prophylactic antibiotics more than three hours after the surgical incision had a twofold increased risk of surgical site infection, whereas those who received antibiotics more than two hours before surgery had a six-fold increased risk [27]. Another study found that when the proper antibiotic was given one hour before the incision, the risk of surgical site infection after total hip arthroplasty was the lowest [28]. In patients having surgery, the preventive regimen should contain an agent that is effective against the most likely infectious organisms, but it does not need to eliminate every possible pathogen. Antibiotics should be chosen depending on the antibiogram of the area. In all clean-contaminated operations and in certain clean procedures where a surgical site infection would be fatal to the patient, antibiotic prophylaxis should be administered. Nevertheless, the microorganisms to be addressed for such prophylaxis are site-specific, most typically Gram-positive cocci, such as staphylococcus, and coverage that is broader or longer than advised has not been demonstrated to lower the incidence of SSIs. According to the current Infectious Diseases Society of America (IDSA) guidelines, narrow-spectrum antibiotic treatment should be chosen based on the kind of operation done. Furthermore, the use of this antibiotic should be limited to one dosage or for no more than 24 hours after surgery [29].

In our study, the duration of the antibiotics prescribed to the patients was not uniform and not as per the standard guidelines. Sixty-three percent (63%) of patients received antibiotics only for a duration of three days; 17% for five days; 15% for seven days, and 5% for more than a week. Also, only 77.9% reported having an adequate dosage of antibiotics as per the hospital administration policy. The term "optimal duration" refers to administering medication for the lowest period necessary to achieve clinical and microbiologic effectiveness. There are a variety of reasons why antimicrobial therapy should be limited to the shortest time possible. They include the possibility for fewer side effects, greater patient adherence, less resistance promotion, and lower costs [30]. The influence of dosage and duration on good treatment results has been widely explored since methods that target only vulnerable organisms might lead to the proliferation of resistant variants. A growing body of data suggests that when using bactericidal chemicals to treat acute infections, giving greater dosages for shorter periods of time (approximately 3 to 5 days) is often as beneficial as, if not better than, giving smaller doses for longer periods of time (10 to 14 days) [31]. The length of dosage will also be addressed from the perspective of reducing a patient's total medication exposure. In another scenario, if the patient uses antibiotics inappropriately, there is a higher chance of antibiotic-resistant bacteria developing, which may become a major clinical problem. Antibiotic dose interruptions that are made too soon might hasten the emergence of resistant microorganisms. In our study, interestingly empirical antibiotic therapy was prescribed upon admission mostly for the diagnosis of urinary tract infections (35.11%) followed by respiratory tract infections (20.22%). This is in contradiction to the study done by Remesh et al., where the antibiotics were mostly prescribed for respiratory infections [25]. Mettler et al. also found that it was the respiratory infections mostly (31.4%) that are diagnosed upon admission required empirical treatment [32].

In this audit, 87.7% of antibiotics were prescribed with brand names and 12.35% with generic names. In a study done by Remesh A, 10.5% were prescribed with generic names [25]. In a study done by Rishi et al., they discovered that using generics was linked with clinical results that were equivalent to using brand-name medicines. These findings might aid in the development of educational programs targeted at boosting patient and provider trust in generic medications' capacity to treat chronic illnesses [33].

As antibiotic resistance spreads, hospitals are now implementing antibiotic stewardship programs, surveillance systems are being built up, access to over-the-counter antibiotics is being restricted, and consumer awareness is being raised.

By providing academic information to clinicians and recommendations based on microbiological evidence, inappropriate antibiotic use has been reduced. The monitoring of resistant strains both in the community and in hospitals provides important information for improved patient care. These results can be used to improve physician education, antibiotic surveillance, and antibiotic prescription trends in our environment.

## Conclusions

By the judicious use of antibiotics, we can reduce the evolution of antibiotic resistance in bacteria and extend the useful life of antibiotics that are still effective. Antibiotic-use patterns must be studied to address complications resulting from many antibiotics. Antibiotics should be chosen depending on the antibiogram of the area. By providing academic information to clinicians and recommendations based on microbiological evidence, inappropriate antibiotic use can be reduced. The monitoring of resistant strains both in the community and in hospitals provides important information for improved patient care. These results can be used to improve physician education, antibiotic surveillance, and antibiotic prescription trends in our environment.

Antibiotic prescribing in hospitals can be improved by optimizing antibiotic selection, re-evaluating antibiotic treatment when diagnostic testing findings are available, and employing the shortest effective period of medication. Prescription audits should be performed on a regular basis to help hospitals rationalize their medicine prescriptions and enhance their overall quality.
